# Up-Regulation of MicroRNA-145 Associates with Lymph Node Metastasis in Colorectal Cancer

**DOI:** 10.1371/journal.pone.0102017

**Published:** 2014-07-14

**Authors:** Wei Yuan, Chenguang Sui, Qian Liu, Wanyan Tang, Huaying An, Jie Ma

**Affiliations:** 1 State Key Laboratory of Molecular Oncology, Cancer Institute & Hospital, Chinese Academy of Medical Sciences, Peking Union Medical College, Beijing, China; 2 Department of Abdominal Surgical Oncology, Cancer Institute & Hospital, Chinese Academy of Medical Sciences, Peking Union Medical College, Beijing, China; Philipps University, Germany

## Abstract

Metastasis is the main cause of mortality in patients with solid tumours. Identifying the exact molecules associated with CRC metastasis may be crucial to understand the process, which might also be translated to the diagnosis and treatment of CRC. In this study, we investigate the association of microRNA expression patterns with the lymph node metastasis of colorectal cancer. Among these candidate miRNAs, the expression of miRNA-145 was significantly related to lymph node metastasis of CRC. Both *in vitro* and *in vivo* study demonstrated that up-regulation of miR-145 could improve the ability of migration and invasion of colorectal cancer cell, while no effect on proliferation was observed. The mechanism of this promotion is associated with the stabilization of Hsp-27, a protein which plays an important role in the promotion of metastasis. These results may be crucial to understanding CRC metastasis and may be translated to the diagnosis and treatment of CRC.

## Introduction

Colorectal cancer (CRC) ranks the third most common tumor and the fourth leading cause of cancer mortality worldwide [1]. Although many achievements have been made in the treatment of CRC in the past decades, the overall survival rate of patients with CRC has marginally changed. Poor prognosis and survival rate are mainly due to metastasis, thus more than one-third of patients with CRC will ultimately develop metastatic diseases [2]. Therefore, identifying the exact molecules associated with CRC metastasis may be crucial to understand the process, which might also be translated to the diagnosis and treatment of CRC.

MicroRNAs (miRNAs) are 21- to 25-nucleotide single-stranded, non-coding RNA molecules that exert their functions by binding to the 3′-untranslated regions of their corresponding mRNA targets [3]. It has been estimated that one-third of the total human genes may be regulated by miRNAs, indicating that miRNAs have pivotal roles in physiological and pathological processes [4]–[5]. A large number of findings show that miRNAs are implicated in human cancers. The inappropriate expression of miRNAs can lead to the aberrant expression of gene products that may contribute to acquisition of the hallmarks of cancer. These observations suggested the function of miRNAs as tumor suppressors or oncogenes [6]–[8].

Recently, convincing evidence showed that a series of miRNAs play crucial roles in CRC metastasis. For example, Asangani et al. identified mir-21 as metastasis promoter in CRC [9]. Liu et al reported that miR-499-5p enhanced cellular invasion and tumor metastasis in CRC by targeting FOXO4 and PDCD4 [10]. Okamoto K, demonstrated that up-regulation of miR-493 during carcinogenesis could prevent liver metastasis in CRC [11]. However, metastasis resulted from a complex cascade of biological processes and the exact molecular mechanisms underlying CRC metastasis are far from being fully understood.

In this study, the miRNA expression profiles in primary CRC lesion with or without lymph node metastasis were analyzed by using a miRNA microarray and quantitative reverse-transcription polymerase chain (qRT-PCR). In this regard, miR-145 was selected as it displayed dramatic up-regulation in CRC with lymph node metastasis in comparison to that without lymph node metastasis. Both *in vitro* and *in vivo* study demonstrated that up-regulation of miR-145 could improve the ability of migration and invasion of colorectal cancer cell. In addition, iTRAQ (isobaric tag for relative and absolute quantification) labeling and 2DLC-ESI-MS/MS (liquid chromatography tandem MS) were employed to identify cellular proteins which were directly or indirectly regulated by miR-145. These results suggested that miR-145 might play an important role in the metastasis of CRC by stabilization of Hsp-27.

## Results

### 1. Different miRNA expression profiles of CRC with or without lymph node metastasis

**Figure 1:**
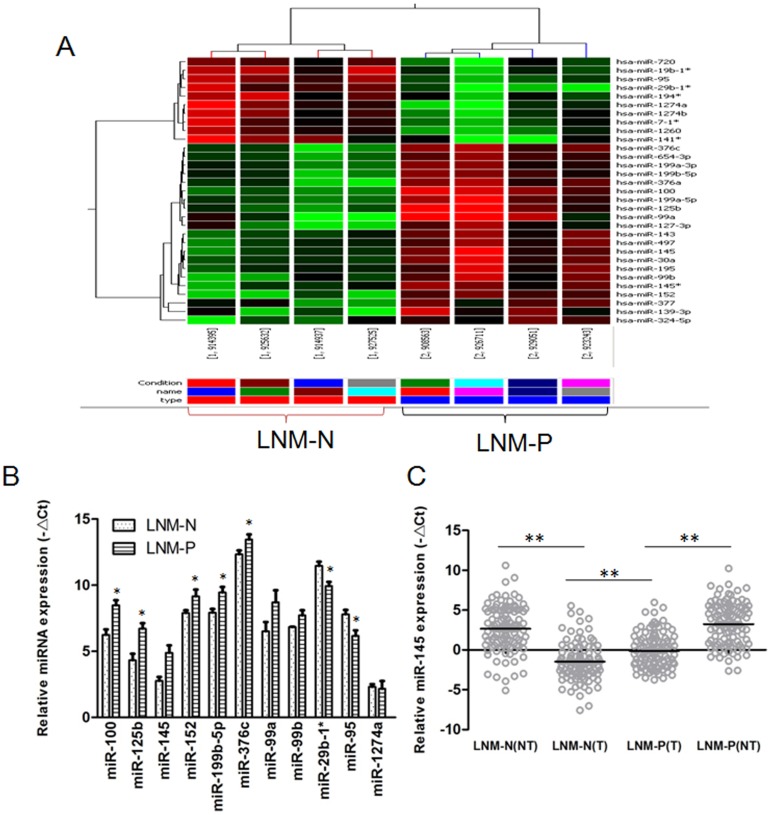
MiRNA expression profiles in CRC with or without lymph node metastasis. A. The certified result of microarray analysis. Hierarchical clustering of 32 significantly dysregulated miRNAs expression profiles in human primary colorectal cancer tissues derived from colorectal cancer patients with (LNM-P, n = 4) or without (LNM-N, n = 4) lymph node metastasis. B.Validation of selected miRNAs predicted to be dysregulated in CRC with or without lymph node metastasis using qRT-PCR in the same tissues used for microarray analysis. Data shown in B is representative of three independent experiments, and presented as fold expression normalized to U6 ± SD (standard deviation).C. QRT-PCR analysis of the relative expression of miR-145 in additional 202 (LNM-N = 99; LNM-P = 103) cases of human CRC tissues, including tumor sample (T) and matching non-tumor tissue sample (NT) from the same patient. Each sample was analyzed in triplicate and normalized to U6. * *P*<0.05, ** *P*<0.01.

To investigate the association of microRNA expression patterns with the lymph node metastasis of colorectal cancer, eight primary colorectal cancer tissues derived from stage II–III colorectal cancer patients with (n = 4) or without (n = 4) lymph node metastasis were collected and the miRNA expression profiles of them were determined using Agilent miRNA microarray. Among the unique 851 human miRNA probe, 32 miRNAs were identified differentially expressed in CRC tissues between lymph node metastasis positive and negative group (*P*<0.05). Twenty one of them were observed considerably increased expression in CRC with lymph node metastasis. On the other hand, 11 of them were underexpressed significantly in lymph node metastasis positive CRC tissues. Unsupervised clustering analysis with these 32 significantly dysregulated miRNAs (21 miRNAs with significant overexpression and 11 miRNAs with significant down-regulation) was able to distinguish the CRCs with or without lymph node metastasis (Fig. 1A).

### 2. Verification of miRNA expression by real-time PCR analysis

To validate the miRNA microarray data, we performed quantitative real-time PCR (qRT-PCR) to analyze the expression level of 11 miRNAs which were the most significantly dysregulated miRNAs or which were not reported its association with metastasis, including miR-99b,-125b,-100,-99a,-152,-199b-5p,-145,-376c,-29b,-95 and -1274a. We examined the expression of miRNAs in the same tissues used for microarray analysis. After normalization with the endogenous control U6, qRT-PCR data confirmed that the expression of 8 miRNAs showed to be consistent with microarray result. Among them, the expression of miRNA-145 displayed the most significant difference between cancer tissues of the two groups (Fig. 1B).

To further confirm the miR-145 expression profile, qRT-PCR analysis was performed in additional 202 CRC samples (99 CRC patients with lymph node metastasis and 103 CRC patients with no lymph node metastasis) (see Table 1 and Table S1). As shown in Fig. 1C, miR-145 was underexpressed in colorectal cancer specimens with or without metastasis compared to adjacent normal tissues respectively, which is consistent with previous reports by others [14]. However, the expression of miR-145 was significantly up-regulated in CRC with lymph node metastasis than that without lymph node metastasis. This change suggested that miR-145 may play an important role in CRC lymph node metastasis.

**Table 1:** The clinicopathologic characteristics of 202 cases of primary CRC patients used in this study.

Clinicopathologic characteristics	Case numbers	%
**Age(Years)**		
** <60**	100	49.5
** >60**	102	50.5
**Gender**		
** Male**	117	57.9
** Female**	85	42.1
**Differentiation**		
** Well**	22	10.9
** Moderate**	161	79.9
** Poor**	19	9.4
**Tumor size**		
** <5**	105	52.0
** >5**	97	48.0
**TNM Stage**		
** I**	35	17.3
** II**	66	32.7
** III**	83	41.1
** IV**	18	8.9
**Lymph node metastasis**		
** Negative**	99	49.0
** Positive**	103	51.0

### 3. Overexpression of miR-145 has no effect on proliferation of HCT-8 cells

**Figure 2:**
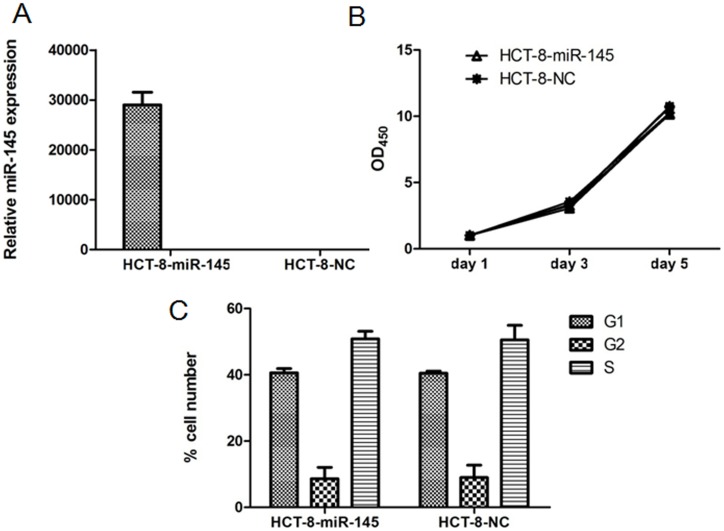
The effect of miR-145 overexpression on the HCT-8 cells. (A) qRT-PCR analysis of miR-145 expression in HCT-8 cells transfected with the lenti-miR-145 expression vector or the miRNA negative control vector using a lentivirus system. (B) Proliferation rates of HCT-8-miR-145 or HCT-8-NC cells detected by CCK-8 assay. (C) Cell cycle analysis of HCT-8-miR-145 or HCT-8-NC cells by Flow cytometry. Data represents average +SD of three independent experiments.

To determine whether the expression of miR-145 affect the biological function of CRC cells, the miR-145 overexpressed model was created in HCT-8 cells using a lentivirus system, which was referred to as HCT-8-miR-145 cells in this paper. The miR-145 levels of HCT-8-miR-145 cells and mock control cells (HCT-8-NC) were determined using qRT-PCR (Fig. 2A). A significant up-regulation of miR-145 in HCT-8-miR-145 cells was observed compared to that in HCT-8-NC cells. To observe the effect of miR-145 on the HCT-8 cells, cell growth rate or cell cycle was evaluated by CCK-8 or FACS assay, respectively. As shown in Fig. 2B and 2C, the proliferation rate or cell cycle of HCT-8-miR-145 cells did not change as compared with HCT-8-NC. This result suggested that overexpression of miR-145 did not significantly influence the proliferation or cell cycle of HCT-8 cells.

### 4. miR-145 promoted CRC migration and invasion *in vitro* and *in vivo*

**Figure 3:**
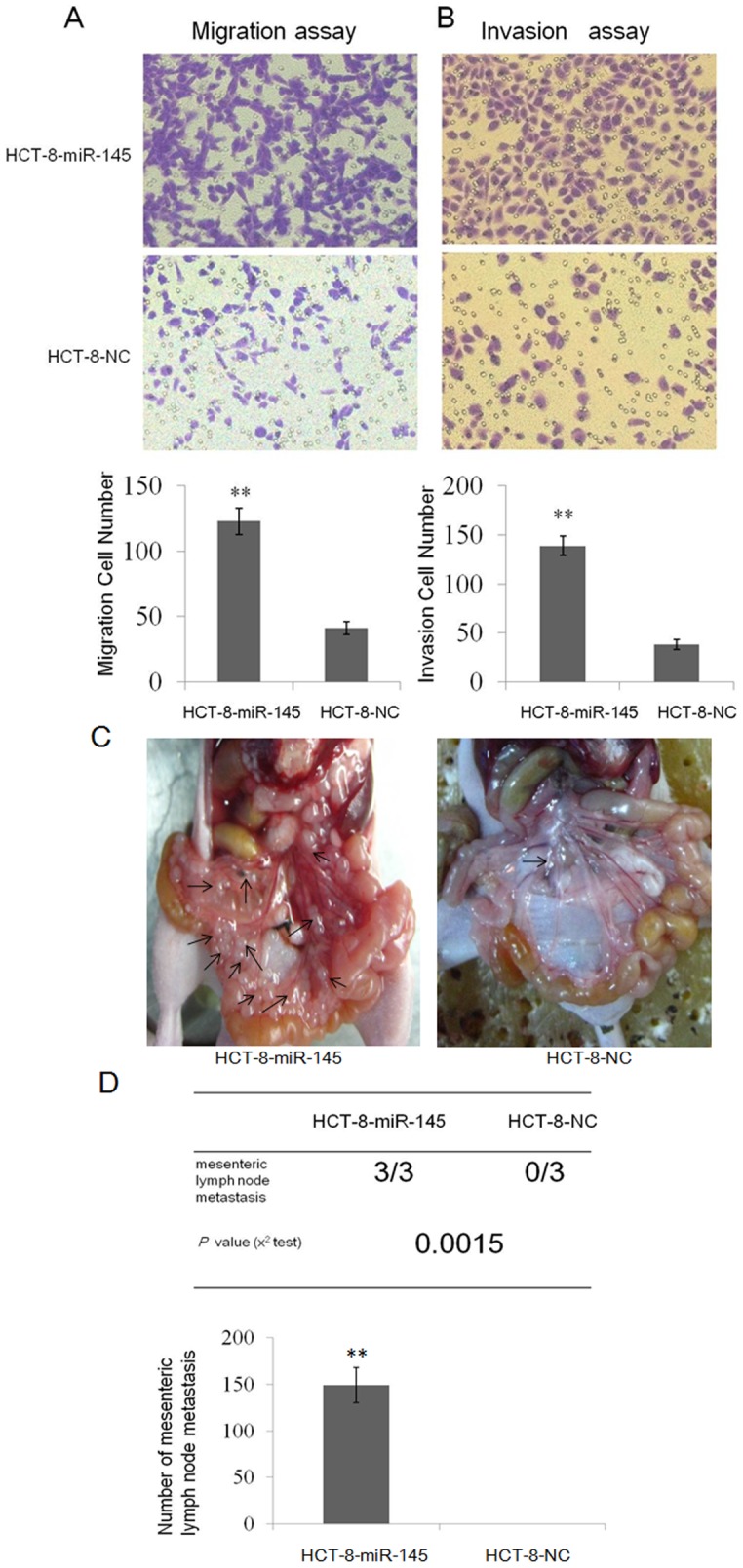
MiR-145 promoted invasion and metastasis of CRC cells *in vitro* and *in vivo.* (A) Migration and invasion (B) assay of HCT-8-miR-145 or HCT-8-NC cells. The images were representatives of at least three independent experiments. Average number of migration cell number per field from at least three independent experiments ± SD is shown by column figure. ** *P*<0.01. (C) Photo images of mesenteric lymph node metastasis from nude mice which was injected into the liver with HCT-8-miR-145 or HCT-8-NC cells followed by surgical suture. Animals were killed 2 weeks post intra-hepatic cell inoculation. (D) Incidence of mesenteric lymph node metastasis in mice (table) and mean number of visible metastatic nodules in mesentery (column figure). ** *P*<0.01.

We subsequently analyzed whether miR-145 contributed to change the migratory mobility of CRC cells. Compared with the mock group, cell migration was significantly increased in HCT-8-miR-145 cells in a transwell cell migration assay (Fig. 3A). A similar result was also observed in a cell invasion assay (Fig. 3B). We also determined the ability of miR-145 to promote migration in other CRC cell lines (SW480 and SW620). As showed in Fig. S1 in File S1, overexpression of miR-145 significantly increased the migratory ability of sw620 cells, whereas no apparent change in sw480 cells (data not shown). Collectively, these results provide strong evidence that up-regulation of miR-145 could promote cell migration and invasion *in vitro*. Because the expression of miR-145 in HCT-8 cells exhibited the lowest expression compared with that in other CRC cells, the rest of the work was focused on this CRC cell line to illustrate typical validation.

To further confirm this notion, an orthotropic transplantation nude mouse model was established to study whether overexpression of miR-145 could promote tumor metastasis *in vivo*. We found no significant difference of the weight or volume of the primary tumors in the liver transplanted by both HCT-8-miR-145 cells and HCT-8-NC cells. We also found that 100% of mice in the HCT-8-miR-145 group had the mesenteric lymph node metastasis and the mean number of metastatic nodules reached 149±15. However, there was none of mice in the HCT-8-NC control group displaying mesenteric lymph node metastasis (Fig. 3C, 3D). Taken together, these results confirmed that high level of miR-145 could promote CRC migration and invasion *in vitro* and *in vivo.*

### 5. Analysis of differentially expressed proteins

The above findings indicated that miR-145 acts as a prometastatic miRNA in CRC. To aim to gain a better understanding of proteins affected either directly or indirectly by miR-145 overexpression, HCT-8-miR-145 and HCT-8-NC cells were lysed, and subjected to iTRAQ labeling, 2DLC-ESI-MS/MS analysis. A total of 1117 distinct proteins were identified and quantified, which were subsequently filtered with manually selected filter exclusion parameters. We took a 1.3 fold change cut-off for the iTRAQ ratio to classify proteins as up or down regulation. This cut-off was applied because several previous iTRAQ studies conducted in our laboratory demonstrated that the technical variation was consistently below 30%, the criterion of cutoff was also accepted by previous research [12]. The proteins were considered up or down-regulated only when their expression ratios (HCT-8-miR-145 cells vs. HCT-8-NC cells) were >1.3 (1×1.3) or <0.77 (1×1.3) AND showed statistically significance.

**Figure 4:**
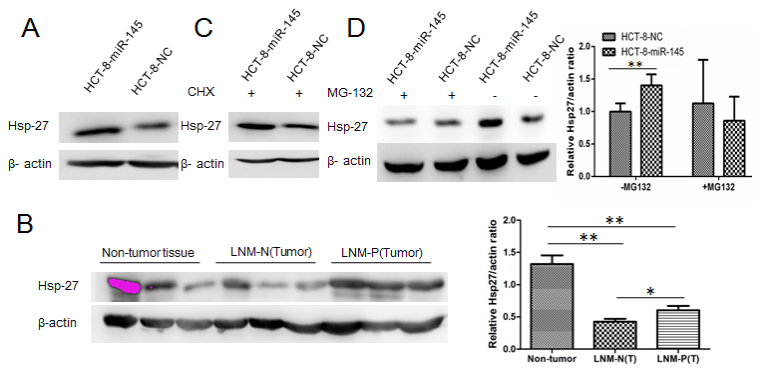
The expression profiles of Hsp-27 were detected in CRC cells or CRC tissues. (A) The expression levels of Hsp-27 were examined in HCT-8-miR-145 or HCT-8-NC cells by western blotting analysis. (B) The expression of Hsp-27 protein was detected in CRC and adjacent normal tissues by western blot assay. The relative Hsp27/actin ratios of individual bands are shown as the mean + SD of values derived from all patient samples (Non-tumor tissue, n = 47; LNM-P tumor tissue, n = 41; LNM-N tumor tissue, n = 43). (C–D) MiR-145 Enhanced Hsp-27 Stability in CRC Cells.HCT-8-miR-145 or HCT-8-NC cells were incubated with the protein synthesis inhibitor cycloheximide (CHX, 0.5 µg/µL) (C) or proteasome inhibitor MG-132 (5 µM) (D) for 24 hours. The level of total Hsp-27 was detected by western blotting analysis. The relative Hsp27/actin ratios of individual bands are shown as the mean ± SD of values normalized to beta-actin.

Thus 13 proteins were screened out as differentially expressed proteins, including 7 significantly up-regulated proteins and 6 remarkably down-regulated proteins (Table S2, S3). Among 13 differentially expressed proteins, the up-regulation of heat shock protein 27 (Hsp-27) was validated using western blotting (Fig. 4A). We therefore chose Hsp-27 for further investigation.

To further validate the association between Hsp-27 protein and miR-145 expression in CRC, we analyzed their expression profile in primary human tissue samples (including 41 CRC with LNM, 43 CRC without LNM, 47 adjacent non-tumor tissue) using western blotting or qRT-PCR respectively. Among the 131 human tissue samples, the expression of Hsp-27 was significantly up-regulated in CRC with lymph node metastasis compared to that without lymph node metastasis. This trend is consistent with miR-145 expression profile. There was a strong, positive correlation (Spearman) between Hsp-27 protein and miR-145 expression (*r* = 0.402; *P*<0.0001) (Fig.4B, Fig. S2 and S3 in File S1). These results indicated that miR-145 is involved, at least partially, in up-regulating of Hsp-27 protein expression.

### 6. Enhancement of Hsp-27 stability by miR-145 in CRC cells

There was no detectable change of Hsp-27 transcriptional level after miR-145 up-regulation (data not shown). Only protein but not mRNA of Hsp-27 was modulated by miR-145, suggesting this regulation is post- transcriptional. To further explore the protein up-regulation of Hsp-27 affected by miR-145, we detected whether miR-145 enhanced the stability of Hsp-27 protein. HCT-8-miR-145 cells and HCT-8-NC cells were treated with either the protein synthesis inhibitor, cycloheximide (CHX), or proteasome inhibitor, MG-132, respectively. As illustrated in Fig.4C and 4D, the enhanced expression of Hsp-27 in miR-145 overexpressed cells was abolished when incubated with MG132. Such phenomenon was not found when incubated with CHX. These results indicated that miR-145 could not influence the protein synthesis of Hsp-27, but could reduce the rate of Hsp-27 degradation, which enhanced its stability.

### 7. Down-regulation of Hsp-27 attenuated the oncogenic effect of miR-145

**Figure 5:**
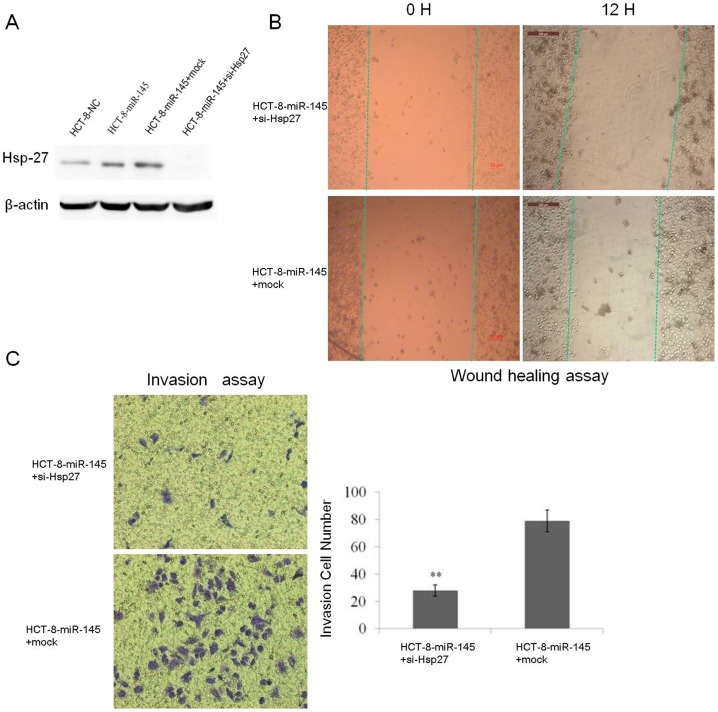
Knockdown of Hsp-27 by siRNA attenuated the prometastatic effect of miR-145. (A) HCT-8-mir-145 (mir-145) or HCT-8-NC (NC) cells were transfected with Hsp-27 siRNA (mir-145+si-hsp27) or a negative control siRNA (mir-145+mock). The expression of Hsp-27 protein was detected by western blot assay. (B) Wound-healing assay to evaluate the effect of Hsp-27 siRNA in HCT-8-miR-145 cells. (C) Knockdown of Hsp-27 by siRNA in HCT-8-miR-145 cells significantly inhibited cell invasion. The images were representatives of at least three independent experiments. Average number of invasion cell number per field from at least three independent experiments ± SD is shown by column figure. ** *P*<0.01.

To verify whether the up-regulation of Hsp-27 contributes to the miR-145-induced motility in CRC cell, a series of assays were carried out *in vitro*. Hsp-27 siRNA or control siRNA was initially transfected into HCT-8-miR-145 cells. The reduction of protein expression of Hsp-27 was confirmed via western blotting (Fig. 5A). Cell migration was then observed using wound healing assay (Fig. 5B). These results showed that the mobility of HCT-8-miR-145 cells transfected with Hsp-27 siRNA was remarkably slower than that of cells transfected with control siRNA. A similar result was also obtained in a cell invasion assay. Transwell-Matrigel penetration assay experiments were performed with HCT-8-miR-145 cells transfected with Hsp-27 siRNA or control siRNA. In comparison to the control, knock down of Hsp-27 inhibited the invasion ability of HCT-8-miR-145 cells (Fig. 5C). The other three different siRNA oligos of Hsp-27 were able to recapitulate similar phenotypes (Fig. S4 in File S1). These results manifested that Hsp-27 was an important downstream mediator during the prometastasis related to miR-145.

## Discussion

Metastasis is the key hallmark of malignance. Although there are many reports of the expression of miR-145 in cancers, the report of the function of miR-145 in the metastasis development of CRC is rarely few. In current study, we investigated the expression of miR-145 in the primary cancer tissue of CRC patients with or without lymph node metastasis. The miR-145 was identified to be underexpressed in CRC specimens with or without lymph node metastasis compared with adjacent normal tissues respectively, which is consistent with previous reports by others [14]–[16]. However, the expression of miR-145 displayed a dramatic up-regulation in CRC with lymph node metastasis, which was out of our expectation. Because to our knowledge, the expression trend of tumor associated genes usually move toward one direction along the development of tumor. To confirm the result of our preliminary observation, the precise expression of miR-145 in the primary CRC tissue of 103 patients with lymph node metastasis and 99 patients without lymph node metastasis was determined using qRT-PCR. Each sample was analyzed in triplicate and normalized against an endogenous control U6. Strict calibration standards and large quantity of tumor specimens used in this study ensured the credibility of our results. We found no significant difference in comparison of miR-145 expression level in adjacent normal tissues between CRC with or without lymph node metastasis. However, a clear association between miR-145 expression and lymphatic metastasis was observed. In these CRC patient samples, miR-145 showed significant higher expression in cancer with lymph node metastasis than those without lymph node metastasis, which further confirmed our array result. Furthermore, as we analyzed data of several other miRNAs, we also observed similar expression profile of decrease, restoration, but not further down-regulation, along with the development of CRC (unpublished data). These observations of other CRC related miRNAs confirmed the accuracy of our results, which suggested that one miRNA maybe display different expression profile in different stages of cancer. Besides stage difference, the expression of miR-145 seems to be dependent on the type of tissue. For example, Sachdeva et al found that the down-regulation of miR-145 was more prominent in CRC than in breast cancer [17]. All of these observations indicated that some miRNAs can be multitasking by interacting with different target genes in various cells and tissues [18].

To investigate the relationship of up-regulation of miR-145 with metastasis of colorectal cancer, miR-145 gain-of-function studies were performed in human CRC cells using lentivirus system. Our results showed that up-regulation of miR-145 could promote CRC migration and invasion *in vitro* and *in vivo,* while no effect on cell growth was observed. These data were inconsistent with some other reports which showed anti-oncogenic role of miR-145 in the metastasis. However, data derived from these observations were either from tissues other than colon [17], [19]–[20]. Arndt et al reported an oncogenic role of miR-145 in CRC, which is in good agreement with our results [21]. These discoveries further confirmed that the function of miR-145 related to cancer development is tissue-type specific.

This study provides the first evidence that miR-145 functions primarily as a prometastatic miRNA in advanced CRC. It is in general that single specific miRNA can regulate multiple target genes, which suggests that single miRNA could carry out a variety of functions by targeting different genes in various cellular contexts [22]. At the early stage of CRC, miR-145 is down-regulated, indicating its target related to cell proliferation. At the advanced stage, high expression of miR-145 might relate to targets against metastasis. MiR-17-5p, a well-investigated miRNA, could target pro- and anti-proliferative genes and act as both an oncogene and a tumor suppressor in different cancers [13], [23]–[24]. MiR-143, another cancer associated miRNA, its expression is always consistent with miR-145 (we did not exam its co-expression with miR-145 in our study), showed multifunction in cancer metastasis [25]. Taken together, these findings support the hypothesis that miR-145 may play a complex function in the development of CRC.

In order to disclose possible effector genes participating in this function, we identified cellular proteins which were directly or indirectly regulated by miR-145 using iTRAQ labeling and 2DLC-ESI-MS/MS. Our analyses showed that Hsp-27 was up-regulated in miR-145 overexpressed CRC cells. Heat shock protein 27 (Hsp-27), an important member of the small Hsp family, is ubiquitously expressed in various cell types and involved in cellular responses for a variety of stresses such as heat shock, hypertonic stress, oxidative stress [26]–[27]. High levels of Hsp-27 have been found to be associated with the metastasis of several tumor types including CRC, prostate cancer, gastric cancer, hepatocellular cancer, head and neck squamous cell cancer [28]–[30]. In particular, it has been proved that increased expression level of Hsp-27 in CRC was related to the lymph node metastasis [31]–[32]. Our results showed that there was a strong, positive correlation between Hsp-27 protein and miR-145 expression in primary human tissue samples, which indicated that miR-145 was involved, at least partially, in up-regulating of Hsp-27 protein expression.

Knockdown of Hsp-27 gene expression by siRNA was found to reverse miR-145-mediated induction of CRC cell migration in our study. The role of miR-145 in the maintenance of high level of Hsp-27 was not through direct gene targeting but stabilization of Hsp-27. Although the role and clinical outcome of Hsp-27 in primary tumors has been well studied and documented [33], its function in metastasis invasion is still unclear. Further investigations to identify the mechanism of Hsp-27 involved in CRC metastasis will further enrich our understanding on the up-regulation of miR-145 in CRC.

In summary, the current study demonstrated that up-regulation of miR-145 contributed to lymph node metastasis of CRC. The mechanism of this contribution associated with the stabilization of Hsp-27, a protein which plays an important role in the promotion of metastasis. Future direction of evaluation of miR-145 should focus on the mechanism study which might lead to its application in metastasis diagnosis and treatment of CRC.

## Materials and Methods

### Clinical samples

The tissue samples analyzed in this study were obtained from 202 patients (117 males and 85 females) undergoing surgical resection for CRC at Cancer Hospital, Chinese Academy of Medical Sciences, Beijing, China. The samples were used with the written informed consents from patients and with the approval of the Chinese Academy of Medical Sciences Cancer Hospital. None of the patients received chemotherapy or radiotherapy prior to surgical resection. The whole samples reflect the natural distribution of clinicopathological characteristics of CRC patients. Resected specimens were histologically examined by hematoxylin and eosin staining. Primary tumor tissues and corresponding adjacent non-tumor tissues were immediately collected after surgical removal and snap-frozen in liquid nitrogen for further use. We divided this patient cohort into two groups. Those with confirmed LNM were termed as lymph node positive (LNP) group and those without detectable LNM were termed the lymph node negative (LNN) group. All cases were reviewed and confirmed by two experienced pathologists. The clinical characteristics of these specimens are shown in Table 1.

### Cell culture

The human CRC cell line HCT-8 was purchased from Institute of Basic Medical Sciences Chinese Academy of Medical Sciences' cell culture center (Beijing, China). The cells were grown in RPMI 1640 supplemented with 10% fetal bovine serum (Gibco, CA), 100 U/ml of penicillin and 100 µg/ml of streptomycin. Cells were incubated at 37°C and supplemented with 5% CO_2_ in a humidified chamber.

### Microarray analysis

Eight primary colorectal cancer tissues derived from stage II–III colorectal cancer patients with (n = 4) or without (n = 4) lymph node metastasis were collected and the expression profiles of miRNA were determined using Agilent miRNA microarray. Briefly, total RNA was extracted from tumor samples using the miRVana miRNA Isolation Kit (Ambion Inc., TX, USA). The quality and quantity of RNA samples were assessed by a 2100 Bioanalyzer using the RNA 6000 Pico LabChip kit (Agilent Technologies, Santa Clara, CA). The microarray contains probes for 851 human miRNAs from the Sanger database v.12.0. The microarray experiments were performed at ShanghaiBio Corporation using Agilent miRNA labeling reagent and Hybridization Kits, Agilent human miRNA array (V2) and Agilent microarray scanner. All original microarray data is deposited in the NCBI GEO database [GSE48074].

### RNA extraction and real-time quantitative reverse transcription–polymerase chain reaction

The miRNA was purified from cultured cells or tissues using the Qiagen miRNeasy Mini Kit. Reverse-transcription reactions were performed using MiScript Reverse Transcription Kit (Qiagen, Germany.). MiScript SYBR Green PCR Kit in combination with miRNA-specific primers (mir-29b-1* cat.no. MS00009289, mir-95 cat.no. MS00010906, mir-100 cat.no. MS00003388, mir-125b cat.no. MS00006629, mir-152 cat.no. MS00003591, mir-376c cat.no. MS00004046, mir-199b cat.no. MS00003731, mir-145 cat.no. MS00003528, mir-99a cat.no. MS00003374, mir-1274a cat.no. MS00014420, mir-99b cat.no. MS00032165, Qiagen, Germany.) were used to detect mature miRNAs on LightCycler 480 (Roche, Basel, Switzerland). The relative expression of miRNA compared with U6 (cat. no. MS00033740, Qiagen, Germany.) was calculated using the -ΔCt method. All qRT-PCR reactions were performed in triplicate.

### Generation of lentivirus to achieve gain of miR-145 function

Lentiviral pGCsil-GFP Vector was used to carrying human pre-miR-145 (miR-145) or nonfunctional control (NC). Lentiviral vector construction and production of high-titer lentiviral particles were made by Genechem biology company. The generated lentiviruses were used to infect HCT-8 for 24–48 h at 1–5 MOI, and the expression levels of miR-145 were determined by qRT-PCR assays. Infected populations exhibiting between 70–90% green fluorescent cells were used for later experimentation.

### Proliferation assay

Cells were grown in RPMI-1640 medium containing 10% fetal serum. 1×10^3^ cells were seeded in flat-bottom 96 well plates and incubated at 37°C in 5% CO2. Cell viability was measured using Cell Counting Kit-8 (Dojindo Laboratories) at day1,day3 and day5. For cell cycle analysis, cells were washed and fixed with ice-cold 75% (v/v) ethanol at −20°C for 2 h, then stained with PI at the concentration of 50 µg/mL in the presence of RNase A (100 µg/mL). DNA content was analyzed by Flow cytometry analysis (Beckman Coulter, USA).

### Cell migration/invasion assays

Cell motility and invasiveness were determined by a 24 well transwell plate (8 µM pore size; Costar), as described previously.^10^ Briefly, for transwell migration assays, 1×10^4^ cells were placed on the top chamber lined with the noncoated membrane. For invasion assays, 3×10^4^ cells were placed on the upper chamber of each insert coated with 200 mg/ml of Matrigel (BD Biosciences, CA, USA).

### *In vivo* metastasis assays

For *in vivo* metastasis assays, 5×10^4^ HCT-8-miR-145 cells or HCT-8-NC cells were injected into the liver of nude mice followed by surgical suture (three in each group, female nu/nu). After 2 weeks, the mice were killed, their livers were dissected, and the mesenteric lymph node metastases were counted. The nude mice were purchased from Vital River (Beijing, China) and raised in a specific pathogen free (SPF) animal laboratory. All experiments involving animals were approved by Chinese Academy of Medical Sciences and Peking Union Medical College Ethical Committee and performed according to the legal requirements.

### iTRAQ labeling and LC-ESI-MS/MS analysis

The cells were washed twice with PBS, collected with a cell scraper, and centrifuged. The pellet was vigorously washed with PBS. After centrifugation at 15,000 rpm for 30 minutes at 4°C, the clarified supernatant was transferred to fresh microtubes and the 2-D Quant Kit (GE Healthcare) was used for the accurate determination of protein concentration in samples followed by analysis in terms of SDS-PAGE. Each sample (100 µg protein) was digested with 0.2 mL of trypsin solution (50 µg/mL) at 37°C. After trypsin digestion, peptides were dried by vacuum centrifugation, reconstituted in 0.5 M TEAB and processed according to the manufacturer's protocol for 8-plex iTRAQ (Applied Biosystems). Briefly, one unit of iTRAQ reagent (defined as the amount of reagent required to label 100 µg of protein) was thawed and reconstituted in 70 µL isopropanol. Peptides from treatment (or disease) and control subgroup were labeled with 114 and 121 iTRAQ tags, respectively, by incubation at room temperature for 2 h. The peptide mixtures were subsequently pooled and dried by vacuum centrifugation. The pooled mixtures of iTRAQ-labeled peptides were fractionated by SCX chromatography (Phenomenex),tandem mass spectrometry (MS/MS) in a LTQ Orbitrap Velos (Thermo fisher) coupled online to the HPLC. The protein experiments were performed at BGI Corporation.

### Inhibition of protein synthesis and proteasome

After incubation with protein synthesis inhibitor Cycloheximide (CHX, 0.5 µg/µl, Beyotime, Shanghai, China) or proteasome inhibitor MG-132 (5 µM, Beyotime, Shanghai, China) for 24 h, cells were collected and processed for western blotting analysis.

### Western blot

Cell proteins were extracted using RIPA buffer (1xPBS, 1% Nonidet P-40, 0.5% sodium deoxycholate, 0.1% sodium dodecyl sulfate (SDS), 1 mM Na3VO4 and 1 mM aprotinin and 1 mM phenylmethylsulfonyl fluoride) and determined using 10% SDS-PAGE. Western blot analysis was performed according to standard procedures as previously described [12]. The expression of β-actin on the same membrane was used as a loading control. Mouse monoclonal anti-Hsp27 antibody (G31) was purchased from Cell Signaling Technology, anti-β-actin Ab (A5411) from Sigma.

### Knockdown of Hsp-27 using small interfering RNA (siRNA)

The siRNA specifically targeting Hsp-27 (Sense: 5′-ACGGUCAAGACCAAGGAUGdTdT-3′; Anti-sense: 5′-CAUCCUUGGUCUUGACCGUdTdT-3′) and control siRNAs were designed as described and were synthesized as 2′-*O*-methyl modification by GenePharma (Shanghai, China) [13]. HCT-8-miR-145 cells were transfected with Hsp-27 siRNA or control siRNAs (200 nM) using Lipofectamine 2000 (Invitrogen) reagent according to the manufacturer's instructions. Lysates or cells were harvested 24 h later and subjected to western blotting or transwell-matrigel penetration assay respectively.

### Wound-healing assay

The cells were grown to confluence, and a wound was made through the monolayer using a p200 pipette tip. After wounding, the culture medium was removed, and cells were washed at least twice to eliminate detached cells. Wound closure was imaged by an inverted microscope at 0, 6, 12 and 24 h after wounding. Three independent experiments were performed.

### Statistical analysis

Statistical analysis was performed using SPSS program version 17.0. *In vitro* and *in vivo* data were evaluated by Student's *t*-test. Unpaired *t*-test were used for analysis in Fig 1B, 1C (LNM-N(T) vs LNM-P(T)) and 4B. Paired *t*-test were used for analysis in Fig 1C (LNM-N(NT) vs LNM-N(T); LNM-P(NT) vs LNM-P(T)),2,3,4D,5,S1 and S4. Data are presented as mean ± standard error of the mean. Error bars are representative of at least three independent experiments. The relationship between miRNA expression levels and various clinicopathologic characteristics were employed using the Mann–Whitney test. Correlations were determined using the Spearman correlation. All *P* values <0.05 were considered significant.

## Supporting Information

Table S1 **Clinical and pathological characteristics of patients.**
(XLS)

Table S2 **Most significant differentially expressed proteins identified in iTRAQ.**
(DOC)

Table S3 **Summary of protein identification and quantification results by iTRAQ.**
(XLS)

File S1 **Supporting Figures. Figure S1,** Migration assay of sw620 or sw480 cells transfected with the lentimiR-145-expression vector or the control vector. **Figure S2,** Hsp-27 protein expression profile in primary human tissue samples (including 41 CRC with LNM, 43 CRC without LNM, 47 adjacent non-tumor tissue) by western blot. **Figure S3,** Correlation between Hsp-27 protein and miR-145 expression in primary human tissue samples (including 41 CRC with LNM, 43 CRC without LNM, 47 adjacent non-tumor tissue). **Figure S4,** Knockdown of Hsp-27 by other three different siRNA oligos in HCT-8-miR-145 cells significantly inhibited cell migration and invasion.
(DOC)
