# Supplementary material for: Up-Regulation of MicroRNA-145 Associates with Lymph Node Metastasis in Colorectal Cancer
Source: PLoS One. 2014 Jul 14;9(7):e102017. doi: 10.1371/journal.pone.0102017 (PMC4096587; doi:10.1371/journal.pone.0102017)

Supplementary Table 2. Most significant differentially expressed proteins identified in iTRAQ.


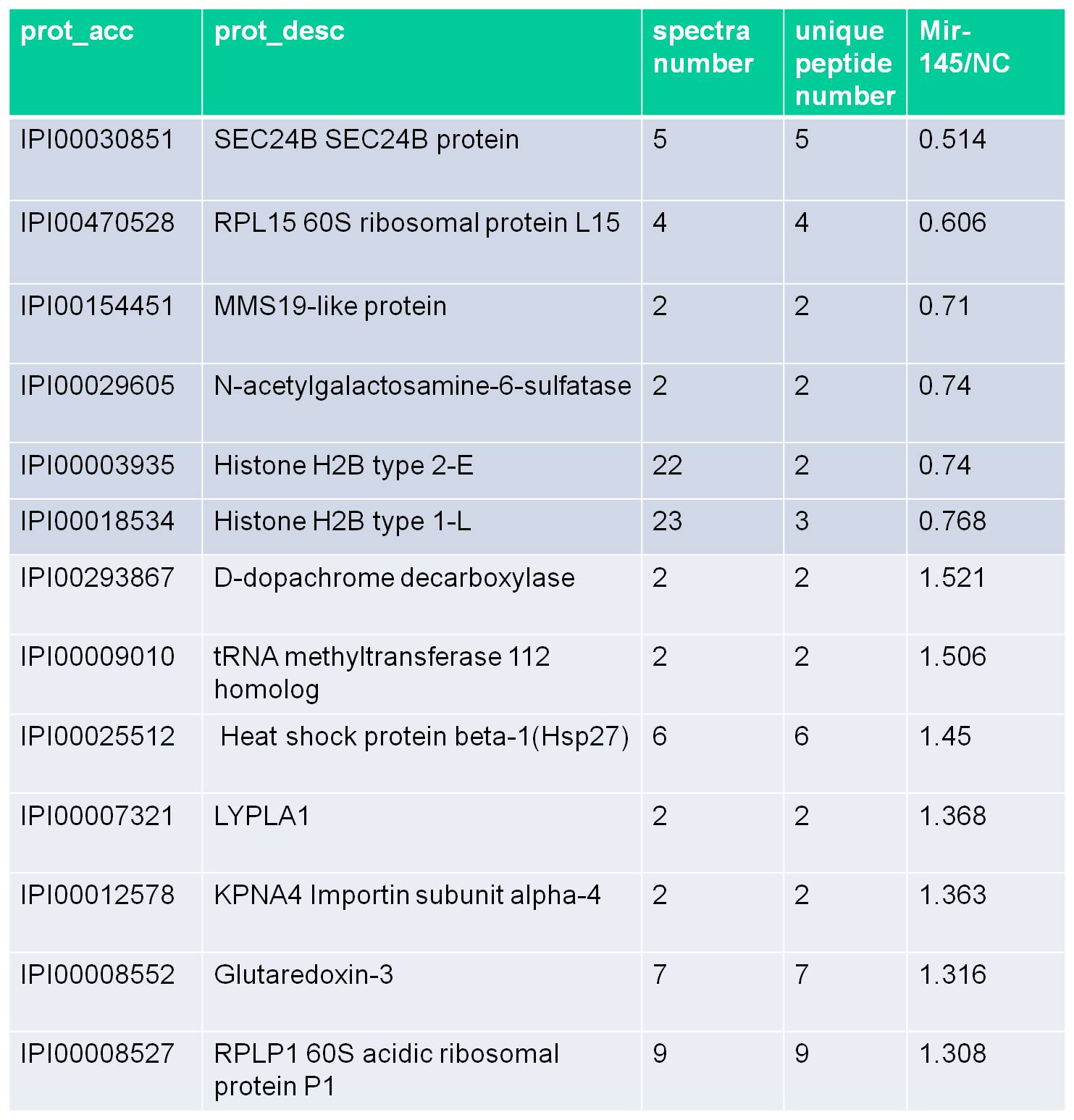

Supplement: Table S2 — Most significant differentially expressed proteins identified in iTRAQ. (DOC) [file pone.0102017.s002.doc]
