# Supplementary material for: Up-Regulation of MicroRNA-145 Associates with Lymph Node Metastasis in Colorectal Cancer
Source: PLoS One. 2014 Jul 14;9(7):e102017. doi: 10.1371/journal.pone.0102017 (PMC4096587; doi:10.1371/journal.pone.0102017)

Supplementary data 4.

Figure s1. Migration assay of sw620 or sw480 cells transfected with the lentimiR-145-expression vector or the control vector. The images were representatives of at least three independent experiments. Average number of migration cell number per field from at least three independent experiments ± SD is shown by column figure. ** *P* < 0.01. NS: no significance.


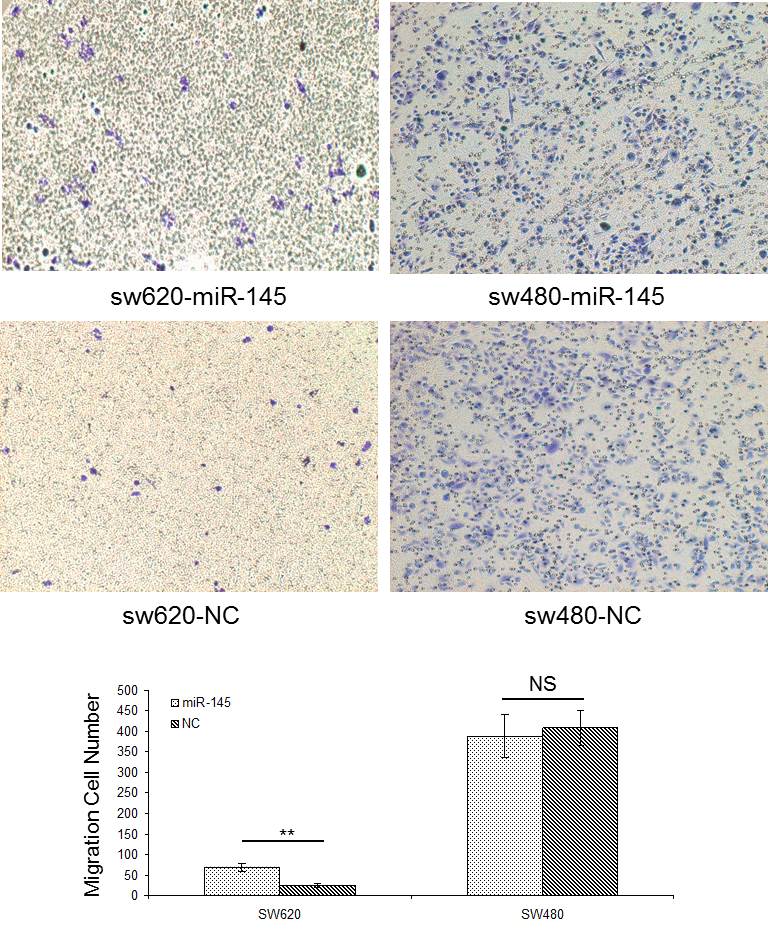


Figure s2. Hsp-27 protein expression profile in primary human tissue samples (including 41 CRC with LNM, 43 CRC without LNM, 47 adjacent non-tumor tissue) by western blot.

C: colorectal cancer; N: adjacent non-tumor tissue


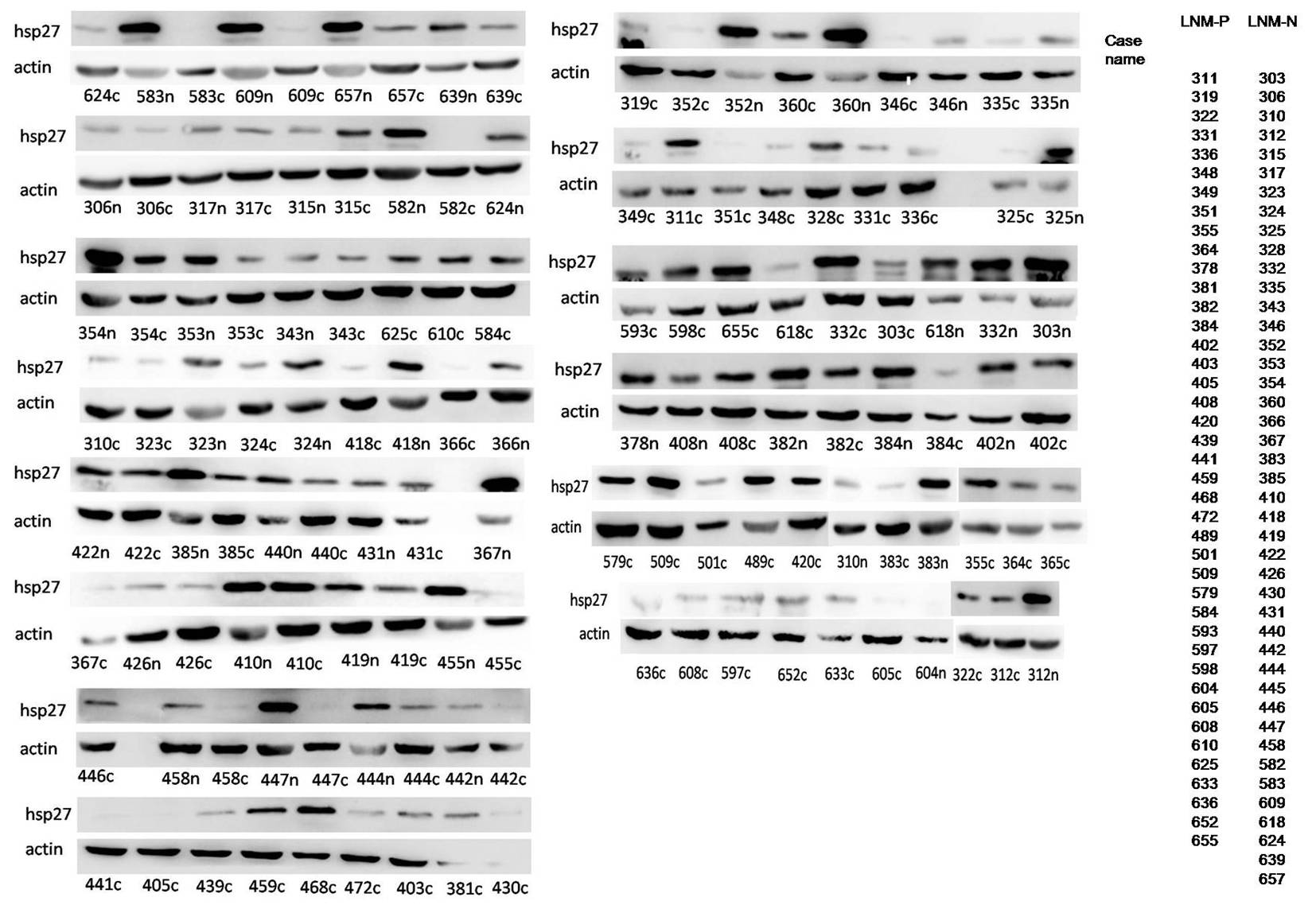


Figure s3. Correlation between Hsp-27 protein and miR-145 expression in primary human tissue samples (including 41 CRC with LNM, 43 CRC without LNM, 47 adjacent non-tumor tissue).


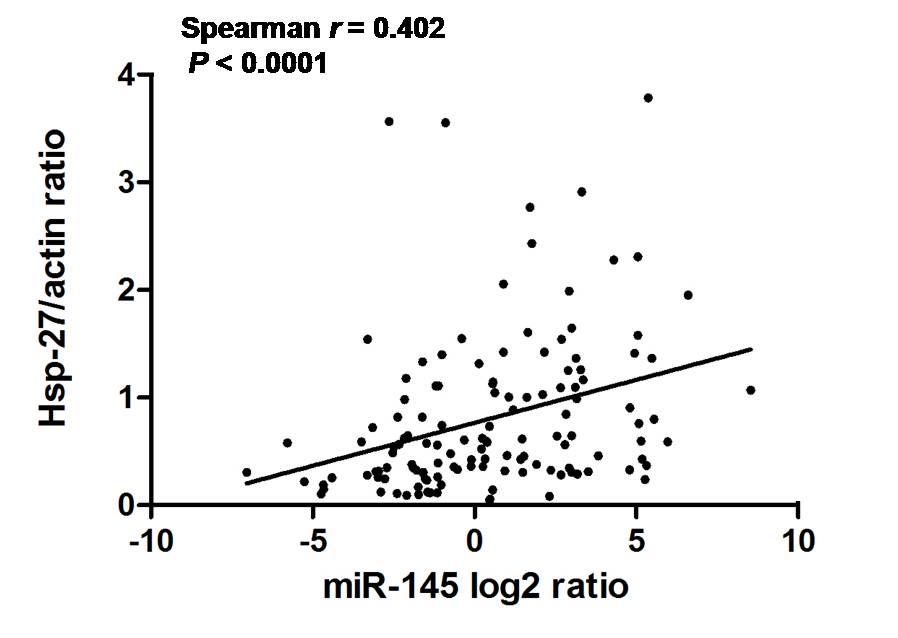


Figure s4. Knockdown of Hsp-27 by other three different siRNA oligos in HCT-8-miR-145 cells significantly inhibited cell migration and invasion. (A)The target sequences of Hsp-27 siRNA were listed. (B) HCT-8-mir-145 cells were transfected with Hsp-27 siRNA or a negative control siRNA (nc). The expression of Hsp-27 protein was detected by western blot assay. (C) Migration and invasion assay of HCT-8-miR-145 cells transfected with Hsp-27 siRNA or a negative control siRNA (nc). The images were representatives of at least three independent experiments. Average number of invasion cell number per field from at least three independent experiments ± SD is shown by column figure. ** *P* < 0.01.


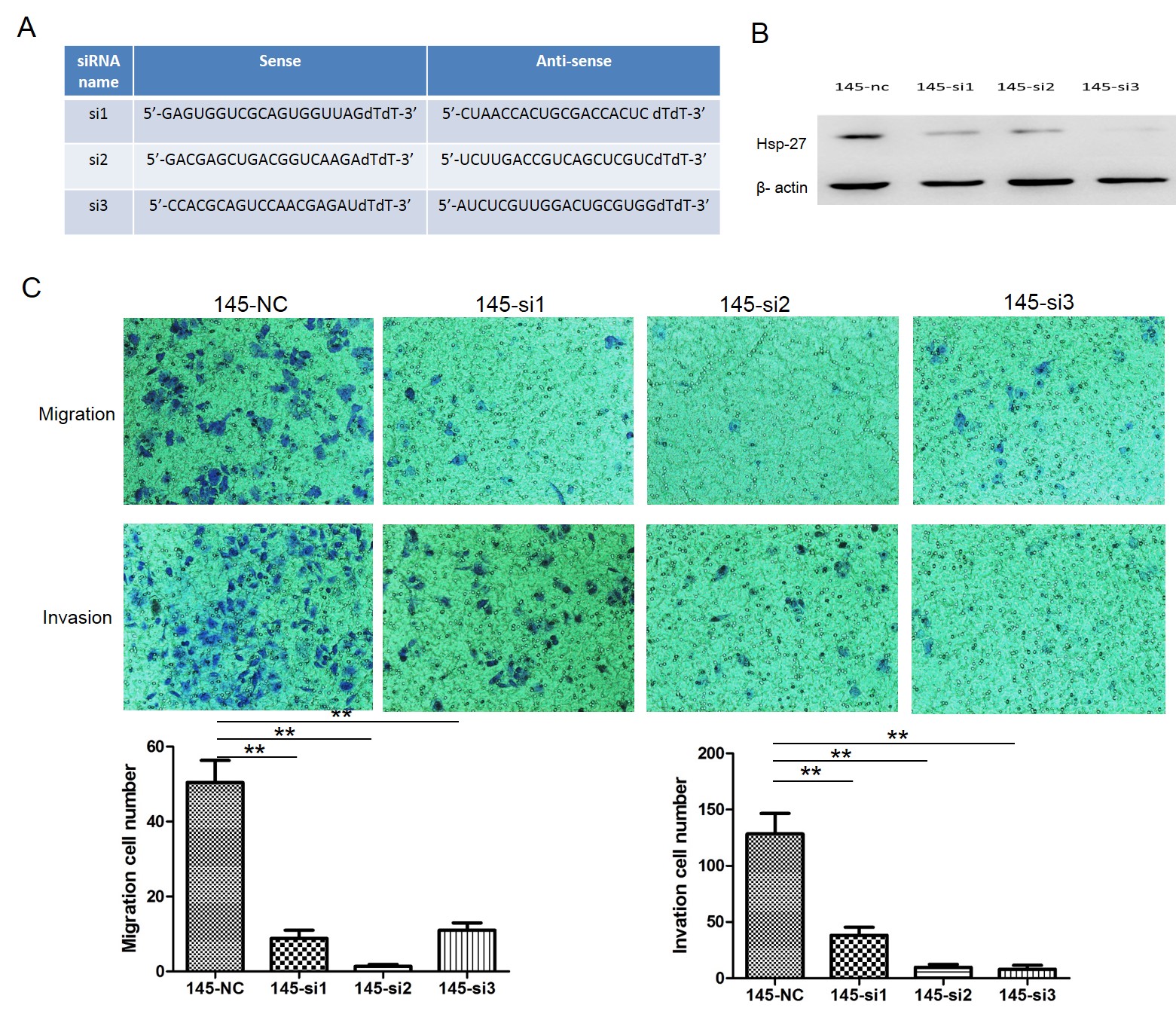

Supplement: File S1 — Supporting Figures. Figure S1, Migration assay of sw620 or sw480 cells transfected with the lentimiR-145-expression vector or the control vector. Figure S2, Hsp-27 protein expression profile in primary human tissue samples (including 41 CRC with LNM, 43 CRC without LNM, 47 adjacent non-tumor tissue) by western blot. Figure S3, Correlation between Hsp-27 protein and miR-145 expression in primary human tissue samples (including 41 CRC with LNM, 43 CRC without LNM, 47 adjacent non-tumor tissue). Figure S4, Knockdown of Hsp-27 by other three different siRNA oligos in HCT-8-miR-145 cells significantly inhibited cell migration and invasion. (DOC) [file pone.0102017.s004.doc]
